# Paper- or Web-Based Questionnaire Invitations as a Method for Data Collection: Cross-Sectional Comparative Study of Differences in Response Rate, Completeness of Data, and Financial Cost

**DOI:** 10.2196/jmir.8353

**Published:** 2018-01-23

**Authors:** Jonas Fynboe Ebert, Linda Huibers, Bo Christensen, Morten Bondo Christensen

**Affiliations:** ^1^ Department of Public Health Research Unit for General Practice Aarhus University Aarhus Denmark; ^2^ Department of Public Health Section for General Medical Practice Aarhus University Aarhus Denmark

**Keywords:** questionnaire study, response rate, completeness of data, financial costs, missing values, selection bias, digital post, digital survey invitation, Web-based questionnaire

## Abstract

**Background:**

Paper questionnaires have traditionally been the first choice for data collection in research. However, declining response rates over the past decade have increased the risk of selection bias in cross-sectional studies. The growing use of the Internet offers new ways of collecting data, but trials using Web-based questionnaires have so far seen mixed results. A secure, online digital mailbox (e-Boks) linked to a civil registration number became mandatory for all Danish citizens in 2014 (exemption granted only in extraordinary cases). Approximately 89% of the Danish population have a digital mailbox, which is used for correspondence with public authorities.

**Objective:**

We aimed to compare response rates, completeness of data, and financial costs for different invitation methods: traditional surface mail and digital mail.

**Methods:**

We designed a cross-sectional comparative study. An invitation to participate in a survey on help-seeking behavior in out-of-hours care was sent to two groups of randomly selected citizens from age groups 30-39 and 50-59 years and parents to those aged 0-4 years using either traditional surface mail (paper group) or digital mail sent to a secure online mailbox (digital group). Costs per respondent were measured by adding up all costs for handling, dispatch, printing, and work salary and then dividing the total figure by the number of respondents. Data completeness was assessed by comparing the number of missing values between the two methods. Socioeconomic variables (age, gender, family income, education duration, immigrant status, and job status) were compared both between respondents and nonrespondents and within these groups to evaluate the degree of selection bias.

**Results:**

A total 3600 citizens were invited in each group; 1303 (36.29%) responded to the digital invitation and 1653 (45.99%) to the paper invitation (difference 9.66%, 95% CI 7.40-11.92). The costs were €1.51 per respondent for the digital group and €15.67 for paper group respondents. Paper questionnaires generally had more missing values; this was significant in five of 17 variables (*P*<.05). Substantial differences were found in the socioeconomic variables between respondents and nonrespondents, whereas only minor differences were seen within the groups of respondents and nonrespondents.

**Conclusions:**

Although we found lower response rates for Web-based invitations, this solution was more cost-effective (by a factor of 10) and had slightly lower numbers of missing values than questionnaires sent with paper invitations. Analyses of socioeconomic variables showed almost no difference between nonrespondents in both groups, which could imply that the lower response rate in the digital group does not necessarily increase the level of selection bias. Invitations to questionnaire studies via digital mail may be an excellent option for collecting research data in the future. This study may serve as the foundational pillar of digital data collection in health care research in Scandinavia and other countries considering implementing similar systems.

## Introduction

The preferred mode for collecting survey data in research has traditionally been the paper questionnaire [1], which is a simple and palpable way of communicating between citizen and researcher. However, in recent years, this way of collecting data has been challenged. Over the last decade, response rates have declined by approximately 1% per year in many countries [1-4]. A low response rate may induce selection bias because respondents may differ systematically from nonrespondents, and the study population will thus not represent the target population [5]. The costs of sending letters by surface mail in the Scandinavian countries have also increased markedly in the last years. For example, a cost increase of 90% was seen in Denmark in 2016 [6,7]. Additionally, longer delivery time (up to 8 days) and fewer post offices may also imply that many Danes now tend to check their physical mailbox less often (M Christensen, email communication, June 5, 2017 and [7,8]).

Data collection by paper questionnaire with traditional surface mail involves several time-consuming and costly steps, such as printing and packing questionnaires and scanning returned questionnaires. A study performed in Denmark in 2013 estimated an average expenditure of €8.62 per respondent, exclusive of motivational costs and researcher time [1]. Filling out a paper questionnaire is a practical and easy method because the respondent only needs a pen and time to participate. Yet, this option also enables the respondent to leave questions unanswered (intentionally or unintentionally), to fill in more answers for one question than allowed, or to check a box outside the intended boundaries. These errors often result in missing values, especially when completed questionnaires are read by a machine, and compromise the data.

The growing use of the Internet has made the Web-based questionnaire an obvious alternative to the paper questionnaire. Web-based studies have been shown to lower the data collection costs [9,10], which is attractive, especially in large population-based surveys [1]. As more and more people have access to the Internet, this has reduced potential variations in the population coverage between paper- and Web-based questionnaires and lowered the risk of selection bias from using the Internet for questionnaire surveys [4]. In 2016, 94% of all Danish citizens had access to a computer and the Internet at home [11]. Furthermore, free public access to the Internet is offered at all Danish libraries, which ensures almost 100% access to the Internet for the entire population.

The Danish government made it mandatory in 2014 for all Danish citizens older than 15 years of age to use a digital mailbox when communicating with public authorities [12]. In exceptional cases, citizens may opt out of this arrangement and receive information by surface mail. The standard arrangement with digital post has two forms: a personal mailbox for each citizen, which is accessed at the public website borger.dk [13], and an electronic mailbox, which is managed by the private company E-boks [14]. The Danish public authorities have thus successfully ensured that the vast majority (89.3%) of the Danish population have access to a secure and low-cost way of receiving letters from many different public authorities. This includes pay slips, invoices, and notices from the national health care services [15].

The availability of digital mail for the majority of Danish citizens makes it possible to study the use of Web-based invitations to scientific studies. To our knowledge, no former study has investigated the impact of being able to reach such a large percentage of the public in terms of data collection.

In this study, we aim to compare the response rates for invitations to a questionnaire-based study sent out by traditional mail and by digital mail. We also aim to compare the completeness of data and the costs of these two ways of collecting data for research.

## Methods

### Design

**Figure 1:**
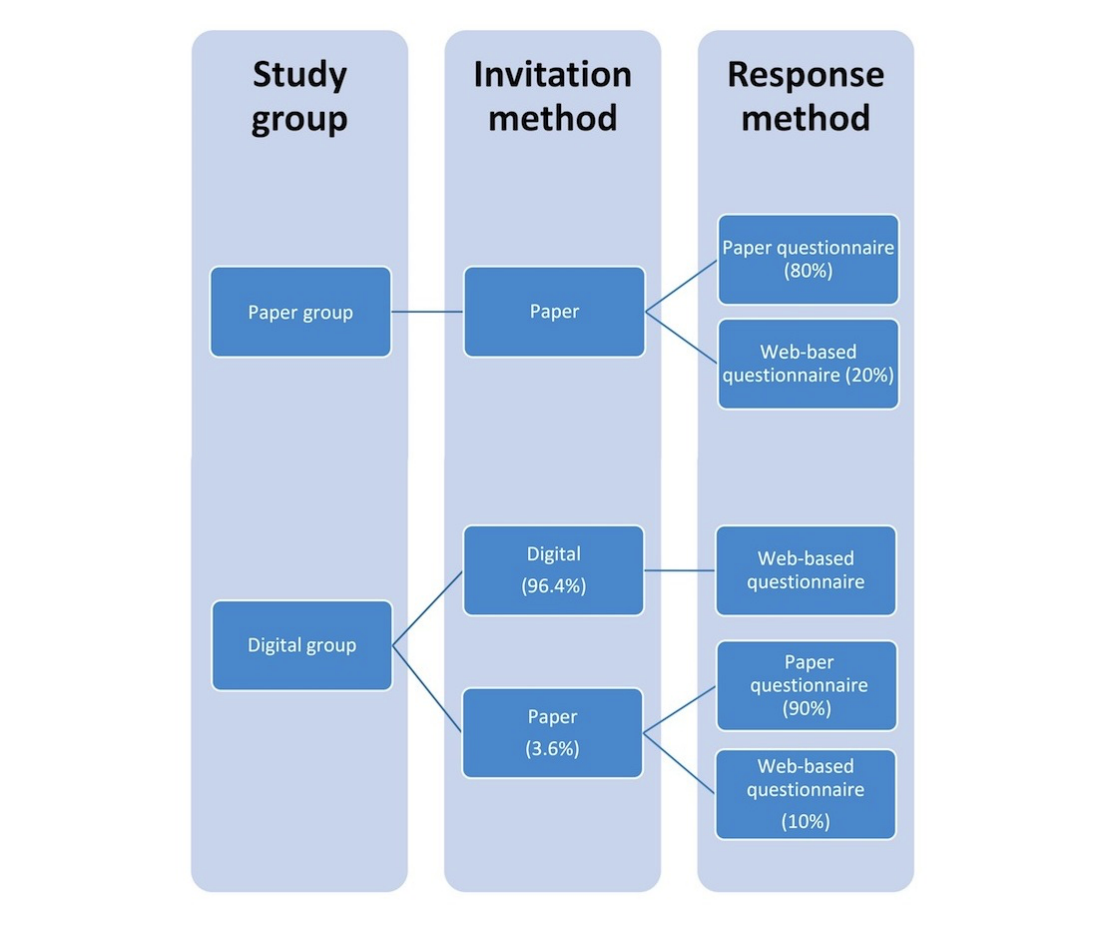
Flowchart of invitation methods and distribution of response methods. Percentages in brackets describe the distribution in the study.

We conducted a cross-sectional comparative study using data obtained from a previous study, which was performed by one of the authors, and a new data collection, which we performed 8 months later. The previous study was based on invitations sent by traditional surface mail (paper group). Respondents could complete an enclosed paper questionnaire or an online questionnaire accessed through a 12-digit code. The new data collection used an invitation sent to an electronic mailbox. This invitation included a unique Web link to a Web-based version of the same questionnaire (digital group). Citizens without an electronic mailbox received the invitation and a paper questionnaire by surface mail. Figure 1 outlines the main components of the two different invitation methods.

### The Questionnaire

The questionnaire had already been developed; it originally formed part of two yet-unpublished studies on citizen help-seeking behavior by Huibers et al (unpublished data, 2017) and Keizer et al (unpublished data, 2017). The aim of this study was to investigate the help-seeking behavior among citizens in need of acute health care during out-of-office hours and the factors related to frequent requests of out-of-hours health care. The questionnaire was sent to parents of children aged 0 to 4 years, to citizens aged 30 to 39 years, and to citizens aged 50 to 59 years. The main part of the questionnaire consisted of six cases presenting well-defined acute health problems; each case was followed by a question on help-seeking behavior with nine multiple response options (including “other”). The parents of young children were presented with different cases than the adult citizens.

Furthermore, 21 questions focused on factors related to help-seeking behavior and background characteristics; these included items from previously validated scales and self-developed questions. The questionnaire regarding children had four additional questions. Questions about factors related to help seeking often included several response options (up to 10 per question) measured by a Likert scale (from a four-point and up to a seven-point scale). Mostly, only one answer was possible. For some questions, the response category “don’t know” and/or “not relevant” was also an option. The background characteristics included a question on age (for which a number should be stated) and several questions with the response option “other” including a free-text field for explanatory comments. Finally, the questionnaire had an extra free-text box for additional comments.

Results from the original study have not yet been published. To our knowledge, this questionnaire is fairly representative of questionnaires used in this field of research. The Web-based questionnaire did not require every question to be answered; this design was chosen to ensure that the method was comparable to the paper questionnaire.

### Inclusion and Exclusion Criteria

In the original study by Huibers et al (unpublished data, 2017), three age groups including 1200 people each were available to compare the two ways of sending out questionnaires: 0 to 4 years (parents), 30 to 39 years, and 50 to 59 years. To get a realistic impression of the all-round response rate, a fourth age group of 70 to 79 years was added. However, this age group was not included in the comparisons of costs and missing values because no information on paper-based data collection was available for this group. We also chose to include 1200 people in each age group because this allowed us to detect a minimum difference in the response rate of 3.8% between the paper group and the digital group if using a significance level of .05 and a power of .9. All citizens included in our study were randomly selected by the Statens Serum Institut, the Danish national institute for health data and disease control, using sex, age, and region to obtain a sample that was representative of the entire country.

The following groups were excluded: citizens living in institutions, citizens with publicly recorded protection against participating in research, and deceased citizens. Furthermore, citizens and siblings of children aged 0 to 4 years who had participated in the paper questionnaire study were excluded from the digital study. Parents of children aged 0 to 4 years were also excluded from the other age groups to ensure that they did not receive two questionnaires. Citizens who returned an empty questionnaire were excluded because of suspicion of disease (eg, dementia or autism) or insufficient language skills. Likewise, citizens who stated that they wished not to participate were registered as nonrespondents. This group of excluded citizens accounted for 0.7% in the paper study and 0.9% in the digital study.

### Data Collection

A total of 89.3% of the Danish population older than 15 years had access to their individual digital mailbox in June 2016. A recent opinion poll showed that 90% of all users reported to read their digital mailbox “always or often” [15,16]. Citizens are linked with the digital mailbox through their unique civil registration number [14]. Citizens younger than 15 years automatically belong under their parents’ digital mailbox; this means that either the mother or both parents receive their digital mail. Citizens can apply for exemption from the digital mailbox in case of cognitive or physical disability, no access to a computer with Internet, language barriers, or if not living in Denmark.

A paper questionnaire containing 27 to 31 questions and subcategories, depending on age group, was sent to the paper group in November 2015 using the traditional method of mailing paper questionnaires (ie, surface mail including a stamped return envelope). After 21 days, a reminder (including a questionnaire and stamped return envelope) was sent by surface mail to nonrespondents.

**Figure 2:**
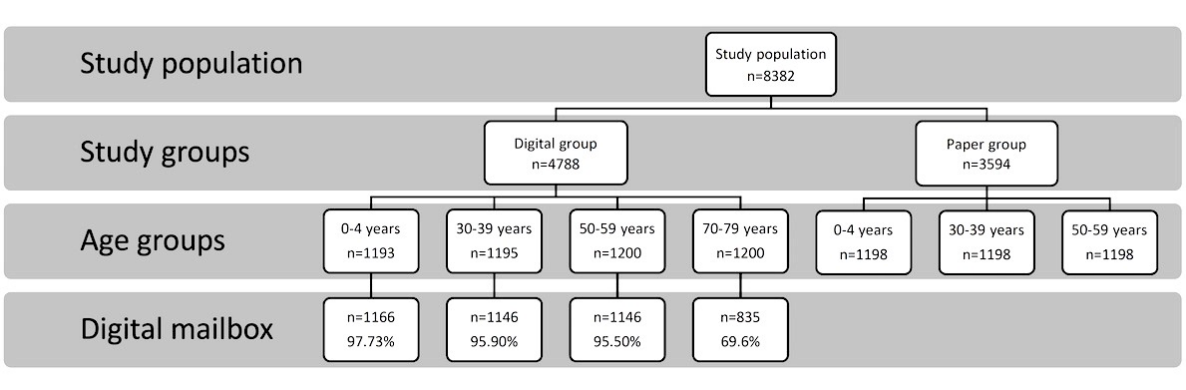
Flowchart of participants and percentage with digital mailbox. The bottom box states the percentage of the age group with access to the digital mailbox (E-boks).

A similar email invitation letter containing a personalized active link to a Web-based version of the questionnaire (using Survey Xact) was sent to the digital mailbox of the digital group in June 2016. The email had a personalized active link that directed the respondent directly to the questionnaire without using any key or password. Nonrespondents received a first reminder after 7 days and a second reminder after 14 days, both using the digital mailbox. The 3.6% of the three youngest age groups (0-4, 30-39, and 50-59 years) in the digital group and the 30.4% of the oldest age group (70-79 years) who did not have a digital mailbox received a paper questionnaire. If they did not respond within 21 days, a reminder was sent by surface mail (see Figure 2). The wording of the invitation was the same for both digital and paper groups, except for the explanation of how to access the Web-based questionnaire. Both groups had the same incentive: to enter a draw to win two cinema tickets.

After the data collection, we received additional data from Statistics Denmark on all citizens included in the study (N=8382). These additional data included family income, immigrant status (born in Denmark or immigrant), education, and job status (employed or unemployed). This information was used to compare respondents and nonrespondents and to compare nonrespondents in the digital group to nonrespondents in the paper group. These comparisons enabled us to assess the level of selection bias. The hypothesis was that no difference between the two groups of nonrespondents would mean that the same degree of selection bias was present in the two methods.

### Outcome Variables

The following primary outcome measures were investigated: difference in response rates, completeness of data, and financial costs. A response was considered valid when a questionnaire was returned by mail or completed online. If a returned questionnaire was blank, it did not count as a response (and was excluded). The completeness of data was assessed by measuring the percentage of missing values in the 17 variables that were considered the most important by the author team who conducted the questionnaire study. To calculate the costs, we measured the time spent on distributing the questionnaires by timing every aspect of the distribution and the data collection for both paper- and Web-based questionnaires. Every expense in the process was registered and summed to calculate a mean cost per respondent.

### Statistical Analyses

When comparing the two distribution groups and respondents to nonrespondents, we used Student *t* test for the variables age and family income. The two-sample test for proportions was used when assessing gender, immigrant status, and job status, and the chi-square test was used to compare educational status. Comparisons were made between and within response groups.

Differences in response rates, overall and within each age strata, were estimated using generalized linear models with ID link and the binomial family. The completeness of data was assessed by calculating the percentages of missing values in the 17 variables that were found most relevant for the study (as described previously) using the two-sample *z* test for proportions. The difference in total costs between the paper group and the digital group was also estimated using generalized linear models with ID link and the binomial family. Only age groups 0 to 4, 30 to 39, and 50 to 59 years were compared in the tables.

We used Stata 14 (StataCorp, College Station, TX, USA) for the statistical analyses.

### Ethical Considerations

The project was approved by the Danish Data Protection Agency (j no 2015-57-0002, AU j no 62908 218). According to Danish law, approval from the Committee on Health Research Ethics of the Central Denmark Region was not needed because the study did not include biomedical intervention.

## Results

The total study population consisted of 8400 people. However, a total of 18 people were excluded: 10 were already included in the paper group, two had an already included sibling, and six had died. More than 96.38% (3458/3588) of the sample had access to a digital mailbox, except in the age group 70 to 79 years in which only 69.58% (835/1200) had access (Figure 2).

### Respondents Versus Nonrespondents

Only a few significant differences were found between the paper group and the digital group within respondents and nonrespondents with respect to age, gender, family income, immigrant status, education and job status in all age groups. Exceptions were job status for respondents in age group 50 to 59 years, family income in age groups 0 to 4 years and 50 to 59 years, age for nonrespondents in age group 30 to 39 years and for respondents in age group 0 to 4 years, and education in age group 50 to 59 years, where a significant difference was found between the digital group and the paper group (*P*<.05) (Table 1).

When comparing respondents to nonrespondents, we saw significant differences (*P*<.05) in age (0-4 years), family income, immigrant status, job status, and education length of more than 15 years (Table 1). In the education variable, 597 missing values were generated; 459 of these concerned immigrants.

Nonrespondents tended to have lower income, to have shorter education, and to be less likely to be employed than respondents. Additionally, more nonrespondents were male.

### Response Rate

The overall response rate in the digital group was 36.31% (1303/3588), almost 10 percentage points lower than in the paper group (45.99%, 1653/3594) (Table 2). In every age group, the response rate was lower in the digital group than in the paper group; the largest difference was seen in the age group 30 to 39 years (paper: 35.81%, 429/1198; digital: 23.18%, 277/1195). In the paper group, 334 of 1653 (20.21%) answered online. In the digital group, 1280 of 1303 (98.23%) answered online.

For the age group 70 to 79 years, the response rate was 50.58% (607/1200). For this age group, no significant difference in response rate was seen between the group who received the invitation digitally (69.58%, 835/1200) and the group who received it on paper (30.42%, 365/1200; difference 1.0%, 95% CI –9.6% to 7.7%).

### Financial Costs

The total costs of collecting data in the digital group was €1969.16 (Table 3). This figure was considerably lower than in the paper group, which amounted to €25,905.28. Although the response rate was lower in the digital group, the costs per respondent were markedly lower than in the paper group (€1.51 vs €15.67). We found that costs related to wages for handling of the digital questionnaires were less than half (43.40%, €926/€2133) of the costs related to handling of paper questionnaires (Multimedia Appendix 1). This lower figure was found even though much more time was spent on handling paper questionnaires, but this work was conducted by assistants who were remunerated at lower wages than the researchers were. We also found that much more time was spent on handling paper questionnaires than on the digital questionnaires (118 hours vs 39 hours, *P*<.001).

### Completeness of Data

Table 4 shows the percentage of missing values in the variables that were found most important by the author team conducting the questionnaire study. The number of missing values was generally lower in the digital group; this was significant in five of 17 variables on a total of nine of 68 occasions.

**Table 1:** Background data for respondents versus nonrespondents, including age, gender, family income, immigrant status, education, and job status (N=7182).

Background data			Invitation method and age range (years)					
			Digital			Paper		
			0-4	30-39	50-59	0-4	30-39	50-59
**Respondents**								
	n		495	277	531	572	429	652
	Age (years), mean (95% CI)		33.3^a^ (32.9, 33.7)	34.5 (34.2, 34.9)	54.5 (54.2, 54.7)	34^a,b^ (33.6, 34.4)	34.7 (34.4, 35.0)	54.2 (54.0, 54.5)
	Gender (male), % (95% CI)		0.2^b^ (–0.2, 0.6)	39.7^a^ (33.9, 45.5)	43.9^a^ (39.6, 48.1)	50.4^b^ (46.2, 54.5)	37.9^a^ (33.3, 42.5)	45.1^a^ (41.3, 49.0)
	Family income (€), mean (95% CI)		34,193 (32,863, 35,523)	35,804^a^ (34,183, 37,424)	46,368^a^ (43,892, 48,844)	35,546^a^ (34,426, 36,666)	35,280^a^ (33,066, 37,494)	44,467^a^ (42,022, 46,912)
	Immigrant status (Danish), % (95% CI)		92.2 (89.8, 94.5)	89.5 (85.9, 93.1)	95.9 (94.2, 97.6)	90.0^a^ (87.5, 92.4)	89.9^a^ (87.1, 92.8)	95.0^a^ (92.7, 96.2)
	**Education (years), % (95% CI)**							
		<10	7.3^a^ (5.2, 9.9)	8.7^a^ (5.8, 12.7)	16.8^a^,^b^ (13.8, 20.3)	8.1^a^ (6.1, 10.7)	8.5^a^ (6.2, 11.7)	20.2^a,b^ (17.3, 23.5)
		10, 15	30.6^a^ (26.7, 34.9)	33.1^a^ (27.7, 39.0)	44.5^a,b^ (40.2, 48.8)	30.4^a^ (26.7, 34.4)	36.3^a^ (31.8, 41.1)	49.0^a,b^ (45.2, 52.8)
		>15	62.1^a^ (57.7, 66.3)	58.3^a^ (52.2, 64.1)	38.7^a,b^ (34.6, 43.0)	61.5^a^ (57.3, 65.5)	55.1^a^ (50.3, 59.9)	30.8^a,b^ (27.4, 34.5)
	Job status (employed), % (95% CI)		80.1^a^ (76.6, 83.6)	84.1^a^ (79.7, 88.4)	82.5^a,b^ (79.2, 85.7)	78.3^a^ (75.0, 81.8)	81.8^a^ (78.2, 85.5)	87.5^a,b^ (84.9, 90.0)
**Nonrespondents**								
	n		698	918	669	626	769	546
	Age (years), mean (95% CI)		32.2^a^ (31.7, 32.6)	34.3 34.1, 34.5)	54.2 (54.0, 54.4)	32.3^a^ (31.8, 32.7)	34.8^b^ (34.6, 35.0)	54.2 (54.0, 54.5)
	Gender (male), % (95% CI)		1.0^b^ (0.2, 1.7)	54.0^a^ (50.1, 57.3)	56.5 (52.7, 60.3)	51.7^b^ (47.8, 55.6)	55.0^a^ (51.5, 58.5)	54.6^a^ (50.4, 58.8)
	Family income (€), mean (95% CI)		32,275^b^ (29,871, 34,678)	31,589^a^ (29,737, 33,441)	40,800^a,b^ (38,678, 42,922)	28,987^a,b^ (27,883, 30,091)	30,481^a^ (29,222, 31,740)	36,913^a,b^ (35,232, 38,594)
	Immigrant status (Danish), % (95% CI)		76.2^a^ (73.0, 79.3)	78.5^a^ (75.9, 81.2)	90.1^a^ (87.8, 92.4)	79.4^a^ (76.2, 82.6)	75.6^a^ (75.6, 81.5)	87.2^a^ (84.4, 90.0)
	**Education (years), % (95% CI)**							
		<10	17.6^a^ (14.7, 20.8)	16.4^a^ (14.0, 19.1)	26.3^a^ (23.0, 29.8)	19.8^a^ (16.7, 23.4)	21.1^a^ (18.1, 24.4)	29.3^a^ (25.5, 33.4)
		10, 15	39.1^a^ (35.3, 43.0)	44.4^a^ (41.0, 47.9)	44.8^a^ (41.0, 48.7)	38.2^a^ (34.1, 42.3)	43.4^a^ (39.7, 47.3)	45.0^a^ (40.7, 49.3)
		>15	43.4^a^ (39.5, 47.3)	39.2^a^ (35.8, 42.6)	28.9^a^ (25.5, 32.5)	42.0^a^ (39.9, 46.3)	35.5^a^ (31.9, 39.2)	25.7^a^ (22.1, 29.7)
	Job status (employed), % (95% CI)		65.2^a^ (61.7, 68.8)	77.2^a^ (73.7, 77.8)	76.7^a^ (73.5, 79.9)	63.4^a^ (59.6, 67.2)	73.9^a^ (70.8, 77.1)	71.8^a^ (68.0, 75.7)

^a^Significant difference (*P*<.05) between respondents and nonrespondents in the same age group and distribution group.

^b^Significant difference (*P*<.05) between paper group and digital group in the same age group and response group. In the age group 0 to 4 years for the paper group, the mean age was calculated on the basis of the mother to ensure compatibility with the digital group. The percent of males in the gender variable for age group 0 to 4 years was low in the digital group because invitations were sent only to the mother, except in cases where the father had sole custody. The paper invitation was sent in the child’s own name directly to the child’s registered postal address.

**Table 2:** Response rates in different age groups for the two ways of collecting data.

Age group^a^	Paper group, n/sent (%)	Digital group, n/sent (%)	Difference, % (95% CI)
0-4 years	572/1198 (47.75)	495/1193 (41.49)	6.25 (2.28-10.23)
30-39 years	429/1198 (35.81)	277/1195 (23.18)	12.63 (9.01-16.25)
50-59 years	652/1198 (54.42)	531/1200 (44.25)	10.17 (6.19-14.16)
All	1653 (45.99)	1303 (36.32)	9.68 (7.41-11.94)

^a^Age group 70 to 79 years was not included in the final response rate.

**Table 3:** Costs (in €) for the two ways of collecting data^a^.

Subject	Paper group (n=1653)	Digital group (n=1303)
Paper questionnaire and envelope	9892	383
Postage	13,815	479
Packaging and registration	768	21
Scanning and coding	1365	17
Digital postage	—	529
Coding	—	394
Handling of digital distribution	—	81
Incentives (draw for 2×2 tickets)	65	65
Total costs	25,905	1969
Costs per respondent	15.67	1.51

^a^Age group 70 to 79 years was not included in this analysis. For further details on costs, see Multimedia Appendix 1.

**Table 4:** Overview of number of missing values in percentage of responses for the two ways of collecting data for different age groups.

Variable		Digital group (years), % missing values				Paper group (years), % missing values			
		0-4 (n=495)	30-39 (n=277)	50-59 (n=531)	Total (n=1303)	0-4 (n=572)	30-39 (n=429)	50-59 (n=652)	Total (n=1653)
**Background characteristics**									
	Age	1.01	0.36	0.56	0.69	1.40	1.40	1.07	1.27
	Gender	1.62	1.08	1.51	1.46	1.75	1.17	1.38	1.45
	Married/cohabiting	1.62	0.72	0.75	1.07	1.75	2.56	2.15^a^	2.12^a^
	Education	0.20	0.72	0.75	0.54	0.70	0.93	0.61	0.73
	Job	0.40	0.36	0.75	0.53	1.22	0.93	0.92	1.03
	Ethnicity	0.81	1.44	3.20	1.99	12.45^a^	1.86	11.99	2.12
**Factors related to help seeking outside office hours**									
	Choice to contact out-of-hours care	5.86	8.66	13.60	9.59	4.20	7.23	11.04	7.68
	Self-efficacy	0.20	1.08	0.56	0.53	0.87	1.40	1.69	1.33^a^
	Anxiety	1.62	0.36	2.26	1.61	1.05	0.93	1.38	1.15
	Social support	0.20	0.36	0.38	0.31	0.87	1.40	1.84^a^	1.39^b^
	Health literacy, navigation	0.40	0.72	1.69	0.99	0.70	0.93	2.15	1.33
	Health literacy, information	0.20	0.00	0.75	0.38	0.52	1.17	1.38	1.03^a^
	Right/barrier	1.21	2.89	2.82	2.22	1.40	2.56	3.53	2.54
	Frequency	0.81	1.81	3.58	2.15	0.87	1.63	1.38^a^	1.27
	Satisfaction with general practitioner	0.40	0.72	1.32	0.84	1.22	0.93	1.23	1.15
	Satisfaction with out-of-hours care	1.41	0.72	2.07	1.53	1.57	1.63	1.84	1.69
	Travel time	0.00	0.00	0.56	0.23	1.05^a^	0.70	1.23	1.03^b^

^a^Statistically significantly more missing values than in the digital group (*P*<.05).

^b^Statistically significantly more missing values than in the digital group (*P*<.01).

## Discussion

### Main Findings

In this questionnaire study, we obtained a significantly lower response rate for invitations sent out by a mandatory secure digital mailbox (36.32%, 1303/3588) than for paper invitations combined with paper questionnaires sent out by surface mail (45.99%, 1653/3594). This difference was seen for all three youngest age groups: parents of children aged 0 to 4 years, citizens aged 30 to 39 years, and citizens aged 50 to 59 years. Citizens aged 70 to 79 years had a high response rate (50.58%, 607/1200).

When exploring the completeness of data, we found that the paper questionnaires generally had more missing values; this was significant in five of 17 variables although variations were found for different age groups.

The costs were markedly lower for the digital method: €1.51 for the digital mailbox versus €15.67 for the paper questionnaire when calculated per respondent.

### Strengths and Limitations

We were able to include a large group of Danish citizens with a secure digital mailbox. This provided us with good power for our analyses, which was a major strength of the study. In addition, we were able to compare nonrespondents in both groups (ie, digital invitation and paper invitation) on socioeconomic variables, which enabled us to evaluate potential selection bias related to applying two different data collection methods. By choosing four age groups that combined represented a substantial part of the population, we were able to explore the general applicability of a digital solution. Still, it is unknown if the response rates for other age groups would be similar to the ones we found.

It was a limitation that the two questionnaires were not sent out at the same time of the year (paper version in November 2015 and digital version in June 2016). Hence, we cannot rule out that some seasonal variation may have occurred. On the one hand, more people are usually ill in November/December, which may have lowered the likelihood of participation and thus lead to an underestimation of the difference in response rates [17]. On the other hand, as mentioned in the Introduction, the general decrease in response rates could have pulled in the other direction. However, we expect this potential variation to be insignificant. In addition, we received some complaints from citizens regarding technical difficulties with the active link in the invitation; this could have had a negative influence on the digital response rates and thus have lead to an overestimation of the difference in response rates.

The questionnaire was not linked to a specific contact with the health care services, which might also have resulted in lower response rates. This also implied that we were unable to make any assumptions about response rates, costs, and completeness of data for specific contacts. Furthermore, we aimed to compare traditional paper invitations and questionnaires with Web-based invitations and questionnaires. However, when sending out paper invitations, it is now common practice to include an option to go online and answer a Web-based version of the questionnaire [1,4]. In our study, 20.21% (334/1653) of the respondents from the paper group used the Web-based option. We chose to include them in the paper group as this realistically reflected the data collection method, but this might also have resulted in lower percentages of missing values and lower overall costs in the paper group compared to complete use of paper questionnaires exclusively. Furthermore, in the calculation of response rates, we also included the small fraction of citizens (in age groups 0-4, 30-39, and 50-59 years) who did not have the digital mailbox and thus received paper questionnaires. The reason was that using only digital invitations for our calculations would not provide us with a precise estimate of the response rate, whereas including the small fraction with no digital mailbox offered a more realistic reflection of the data collection method. The response rate would have been 37% (one percent point higher) if we had included only the digital invitations.

### Interpretation of Results

Sending out paper questionnaires is a costly and time-consuming process [1,4]. Several studies have compared sending out invitations to paper-based and Web-based questionnaires. The overall trend shows little or no difference in the response rates between the two different data collection modes, with response rates ranging from 53% to 92% for the Web-based method and from 56% to 92% for the paper-based method [1,4,18-20]. However, our study found a significant difference in the response rates when comparing paper-based and Web-based methods (46%, 1653/3594 vs 36%, 1303/3588). The biggest difference in response rates was seen in the age group 30 to 39 years (12.7%), which is significantly higher (*P*<.001) than seen for parents of the age group 0 to 4 years (6.1%). An interesting finding was that the age group 30 to 39 years, with a mean age of 34.5 (SD 3.0) years, had significantly lower response rates than the parents of age group 0 to 4 years, with a mean age of 33.3 (SD 4.6) years (23.18%, 277/1195 vs 41.49%, 495/1193, *P*<.001). We believe that this was unrelated to sending out invitations only to the mothers of children aged 0 to 4 years in the digital group because approximately half of the respondents for children aged 0 to 4 years in the paper group were male. Studies using questionnaires concerning a specific contact generally get higher response rates [4]. This could be part of the explanation for the lower response rate in our study compared to the ones found in otherwise comparable studies [1,4,18-20].

Web-based questionnaires have repeatedly been proven to lower data collection costs [1,9,10]. Our study supports these findings as we saw a cost difference by a factor of 10 (€15.67 vs €1.51). In our calculations, we have taken into account the time it takes to handle paper and digital questionnaires. As the costs relating to wages in the handling of digital questionnaires are less than half (43%) the amount relating to the handling of paper questionnaires, this emphasizes that far more time is spent on handling paper questionnaires than digital questionnaires (118 hours vs 39 hours, *P*<.001). If we consider the completeness of the responses, earlier studies have shown that Web-based questionnaires have fewer missing values [21,22]. We looked at the percentage of missing values in a predefined number of variables, and our findings confirm that the completeness of data is higher when the invitation and response method is Web-based.

Van Gelder et al [4] stated in 2010, “Recent studies have already shown that respondents to Web-based questionnaires are comparable to those responding to traditional modes of data collection in terms of age, gender, income, education....” This is also in line with our findings. Furthermore, our analyses indicated that nonrespondents in the paper group and in the digital group did not differ from each other, except for family income, which implies that selection bias might not be a bigger problem in Web-based data collection than in traditional paper-based data collection [2,4].

Moreover, the older age groups tend to have less experience with using computers and the Internet [20], which is shown in the fact that only approximately 70% of this age group had access to a digital mailbox. However, in the near future, we expect to see an increasing use of the Internet in the older age groups, which could facilitate the use of electronic invitations and questionnaires and make it the new way of collecting data. In addition, we expect easier access to the digital mailbox, and the population will become more accustomed to checking their digital mailbox regularly, which might also help improve response rates in questionnaire studies.

### Implications for Practice and Future Studies

Using digital mail as a new way of sending out questionnaires could be the future approach in questionnaire-based research. Because of the reduced costs, sending out more questionnaires for the same amount of money as that required for a paper-based trial could increase the power of a study. Potential selection bias remains an issue as in other questionnaire-based studies, although our results show that the direction of selection bias is similar for both methods of collecting data.

We chose not to include expenses for software to handle the Web-based questionnaires and hardware to handle the paper responses as this was available at our research unit as part of a university institution. Nevertheless, if an independent research group was to buy licenses to conduct Web-based surveys, these would entail considerable costs, which should be considered in the planning.

Citizens are increasingly flooded with online invitations to participate in evaluations and questionnaire surveys. It is important to develop standards when using digital mail for scientific research to maintain this method as a valid way of collecting data in the years to come. One option could be to use only digital mail when inviting citizens to a health-related questionnaire study regarding a specific contact with the health care system. It is our intention to test this in an upcoming study in the Danish out-of-hours primary care setting at the end of 2017 [23]. Future studies could compare response rates for different types of questionnaires when using this option. Another way of achieving acceptable response rates could be to use consumer panels, such as the global network operated by Kantar Active in 90 countries under different names (eg TNS Nipo in the Netherlands and TNS Gallup in Denmark), for which expenses saved are used to pay respondents [24].

### Conclusion

A digital platform offering a secure communication system and targeting the individual citizen combined with easily accessible Web-based questionnaires to be completed on a mobile phone, tablet, or computer seems to be a low-cost option in future survey studies. Such a digital solution also appears to give higher completeness of data compared to data collection by paper invitations combined with paper questionnaires sent by surface mail. Lower response rates (especially in the younger age groups) could be a problem. Still, our findings suggest that the two methods exhibit similar degrees of selection bias for socioeconomic variables.

The secure digital post solution used in our study (e-Boks) is now available in Denmark, Norway, and Sweden, and the system has more than 12 million users in these countries. In the near future, we could see a massive shift from paper to digital data collection in questionnaire research. This project could serve as the foundational pillar of digital data collection and help us obtain a better understanding of the feasibility of this method in future health care research.

## Appendix

Multimedia Appendix 1 Complete overview of financial costs.
